# Supplementary material for: Bidirectional thermo-regulating hydrogel composite for autonomic thermal homeostasis
Source: Nat Commun. 2023 May 26;14:3049. doi: 10.1038/s41467-023-38779-w (PMC10220006; doi:10.1038/s41467-023-38779-w)
Supplement: Supplementary file 1 — Supplementary Information [file 41467_2023_38779_MOESM1_ESM.pdf]

# Supplementary Information

# Bidirectional Thermo-regulating Hydrogel Composite for Autonomic Thermal Homeostasis

*Gyeongsuk Park[1,3], Hyunmin Park[1,3], Junyong Seo[2], Jun Chang Yang[1], Min Kim[1], Bong Jae Lee[2] and Steve Park[1]*

[1] Department of Materials Science and Engineering, Korea Advanced Institute of Science and Technology, Daejeon, 34141, Republic of Korea

[2] Department of Mechanical Engineering, Korea Advanced Institute of Science and Technology, Daejeon, 34141, Republic of Korea

[3]These authors contributed equally: G. Park, H. Park

Correspondence and requests for materials should be addressed to S.P. (email: stevepark@kaist.ac.kr)

**Supplementary Note 1. Numerical calculation of emissive heat flux from the hydrogels.**

The emissive heat flux $E(T)$ from the combined system (i.e. hydrogels loaded on a skin model) was calculated with the measured emissivity under the assumption that the hemispherical emissivity is the same as the normal component due to excessive volumetric scattering effects by silver particles. The skin model was made of Ecoflex, which has similar thermal conductivity and emissivity to actual human skin. The measured emissivity of the system integrates the multiple aspects of heat transfer mechanisms, which occur inside and boundaries of the hydrogels and the skin model. Accordingly, the total amount of emitted heat flux from the system can be calculated as following equation[1].

$$E(T) = \int_{7.5\ \mu\text{m}}^{14.0\ \mu\text{m}} \varepsilon_\lambda E_b(T,\ \lambda) d\lambda\ +\ \int_{3\ \mu\text{m}}^{5\ \mu\text{m}} \varepsilon_\lambda E_b(T,\ \lambda) d\lambda$$

where $E_b(T,\lambda)$ is the spectral emissive power of a blackbody following Planck's distribution at temperature[2], T. The spectral ranges of integration were chosen as 3 to 5 $\mu m$ and 7.5 to 14.0 $\mu m$ to match with the perception ranges of infrared radiation (IR) camera for quantitative comparison between IR camera images. The spectral emissive power of a blackbody $[E_b(T,\lambda)]$ follows Planck's distribution is

$$E_b(\lambda, T) = \frac{C_1}{\lambda^5\left(e^{\frac{C_2}{\lambda T}} - 1\right)}.$$

with the wavelength, $\lambda$ (μm), a surface temperature, $T$ (K), first and second radiation constants $C_1 = 2\pi h c_o^2 = 3.742 \times 10^8\ \text{W} \cdot \mu\text{m}^4/\text{m}^2$, and $C_2 = hc_0/k_B = 1.439 \times 10^4$ $\mu\text{m} \cdot \text{K}$, respectively.

**Supplementary Note 2. Scattering efficiency analysis of diversely shaped silver nano to micro-sized particles.**

As described in Supplementary Figure 9, when electromagnetic resonance of a metallic nano to micro-sized particles are excited by incoming electromagnetic wave (e.g., localized surface plasmon polariton) [3], the particles would interact with the light not only directly incident to the particle but also passing indirectly to the particle. Consequently, the particle scatters or absorbs much larger energy than the incident light only on the cross-sectional area of the particle (i.e. $Q_{sca} > 1$). Additionally, the electromagnetic interaction of the particle depends on the size, shape, materials of the particle, and the incident wavelength, thus, a scattering efficiency spectrum of a given particle should be carefully calculated. Nano-sized silver spheres have a negligible scattering effect at the mid-infrared spectral region due to $Q_{sca} \propto 1/\lambda^4$ in the Raleigh scattering regime. Scattering efficiency of a small particle can be calculated by analytical method or numerical method. For a spherical particle, Mie-scattering theory[4] was applied to calculate its scattering efficiency spectrum. For a wire-shaped particle (i.e., rod shape), open-source boundary element method software, MNPBEM[5] was used. The method divides the surface of particle into elements and numerically obtain the scattering and absorption responses element by element. Especially, the silver micro/nanowire interacts with an incident light differently with respect to its orientation. Therefore, scattering efficiency was obtained by averaging responses along three principal directions of polarization (i.e., one polarization in axial incidence, and two polarizations in radial incidence, Supplementary Fig. 16). The geometrical cross-sectional area of particle was calculated as $\pi R^2$, where $R = \sqrt[3]{3/4 \times (\text{Volume of a particle})/\pi}$ is an effective radius of particle[6]. For the material property of silver, Lorentz-Drude oscillator model[7] was used in this work.

**Supplementary Note 3. Scattering effect of porosity and pore size in ATHH**

The Scattering efficiency of nano to micro-sized materials in ATHH was previously calculated by the boundary element method software (MNPBEM[5]) in Supplementary Note 2. To investigate a structural affect, We also conducted theoretical estimation of the emission characteristic of ATHH based on both porosity and pore size. First, the components of ATHH which are PNIPAm and PPy were measured through FT-IR spectrometer with an integrating sphere. Transmittance and reflectance of components were fitted using Lorentz-Drude model[8] as permittivity for verifiying thermal scattering effect. After obtaining the permittivity spectra of components, permittivity has been determined using effective medium theory[9]. Finally porous structure of ATHH was modelled utilizing simple Monte-Carlo simulation[10] by treating pores as simple air-filled microparticles. Emissivity in Supplementary Figure 19 where fv is the porosity stands for the theraml scattering effect and heat trapping ability. High porosity in ATHH blocks an emissive heat into the outside, which causes considerable thermal scattering in the ATHH medium, and reults as a low emissivity. Although emissivity is inversly proportional to the porosity, the size of the pores confirmed that it is not directly related to the value of the emissivity.

**Supplementary Note 4. Calculation of insulated heat.**

The physical meaning of the spectra obtained by a differential scanning caloriometer (DSC) used to measure the LCST of hydrogel is the required energy for phase transition according to temperature change. Based on this, we obtained the cumulative distribution function of the DSC data to determine the degree of progression of phase transition and the area of the opening of the pattern as the temperature changes. Firstly, insulated heat of solid PNIPAm and ATHH was calculated as the difference between the heat flux of each sample on skin model (emissivity of skin model≈1) presented in Figure 2c and the heat flux of the skin model at the same temperature with sample. The difference represents heat trapped by the sample placed on the skin model when the supplied heat is radiated from the skin model. In case of patterned samples, we estimated their heat flux by correcting heat flux of solid samples using the cumulative distribution function to apply the area of the opening of the pattern according to temperature change.

**Supplementary Table 1. Operating principles and key elements of the thermal management systems.** The list contains the characteristics of various thermal management systems studied in previously reported papers[11-15].

| | Fabric | | | Joule heating device[14] | Thermoelectric device[15] | **This work** |
|---|---|---|---|---|---|---|
| | Heating[11] | Cooling[12] | Dual Mode[13] | | | |
| Operation Mechanism | Radiative manipulation<br>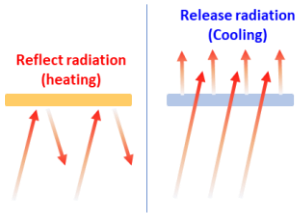<br> | | | Applying electric poentrial<br>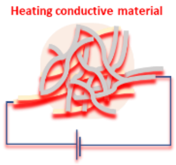<br> | Applying electric potential<br>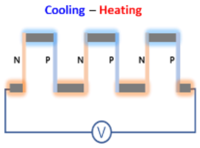<br> | Autonomous actuation<br>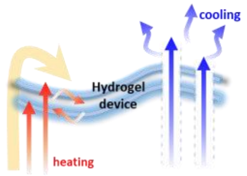<br> |
| Bi-directionality | X | X | O | X | O | O |
| Reversible autonomic operation | X | X | X | X | X | O |
| Conformability | Δ | Δ | Δ | Δ | Δ | O |

**Supplementary Table 2. The comparison table with other materials and textiles for heating effect.** The list contains operation mechanism, value of optical properties, and temperature deviation from the initial state which were studied in previously reported papers [16-22].

| **Material / Textile** | **Optical property (R / T)** | **Value** | **Temperature deviation (from the initial, °C)** | **Reference** |
|---|---|---|---|---|
| Omni-heat (R) | Reflectance | ~41% (IR) | 4.4°C (33°C) | 16, 17 |
| Mylar blanket (space blanket) | Reflectance | ~95% (IR) | 6.9°C (33°C) | 16 |
| Acrylic | Reflectance | ~23% (IR) | 2.9°C (35°C) | 18 |
| Cotton | Reflectance | ~13% (IR) | ~1°C (35°C) | 19, 20 |
| ZnO/Cotton | Transmittance / Reflectance | ~10% (UV) / ~ 45% (UV) | 2.7°C ~ 4.2°C (37.1°C) | 21 |
| PNIPAm/BN-OH | Transmittance | ~50% (IR) | 2.5~2.9°C (29.8~30.3°C) | 22 |
| **This work** | Transmittance | ~1% (IR) | 6°C (35°C) | |

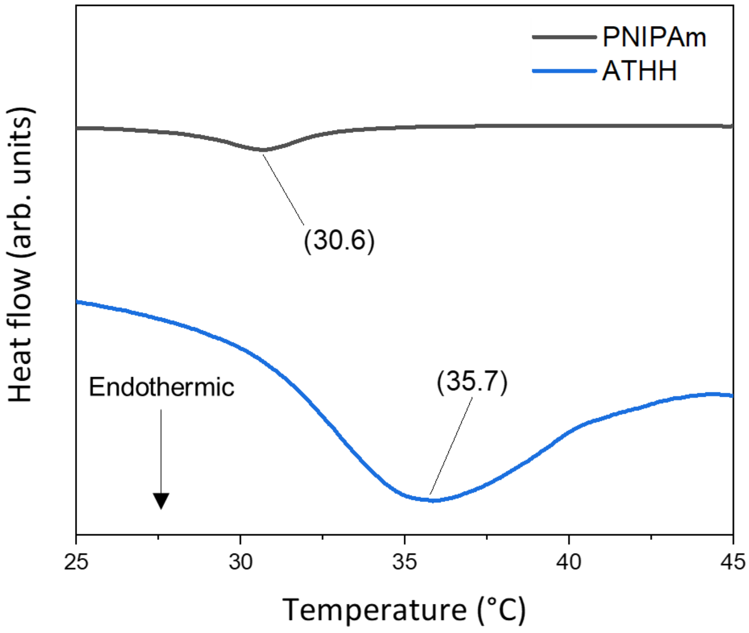


**Supplementary Figure 1. Differential Scanning Calorimeter (DSC) data of ATHH and PNIPAm.** The temperature at a peak of heat flow in DSC data means LCST of hydrogel. PNIPAm shows LCST at 30.6°C, whereas, LCST of ATHH is 35.7°C closer to normal human body temperature of 36.5°C.

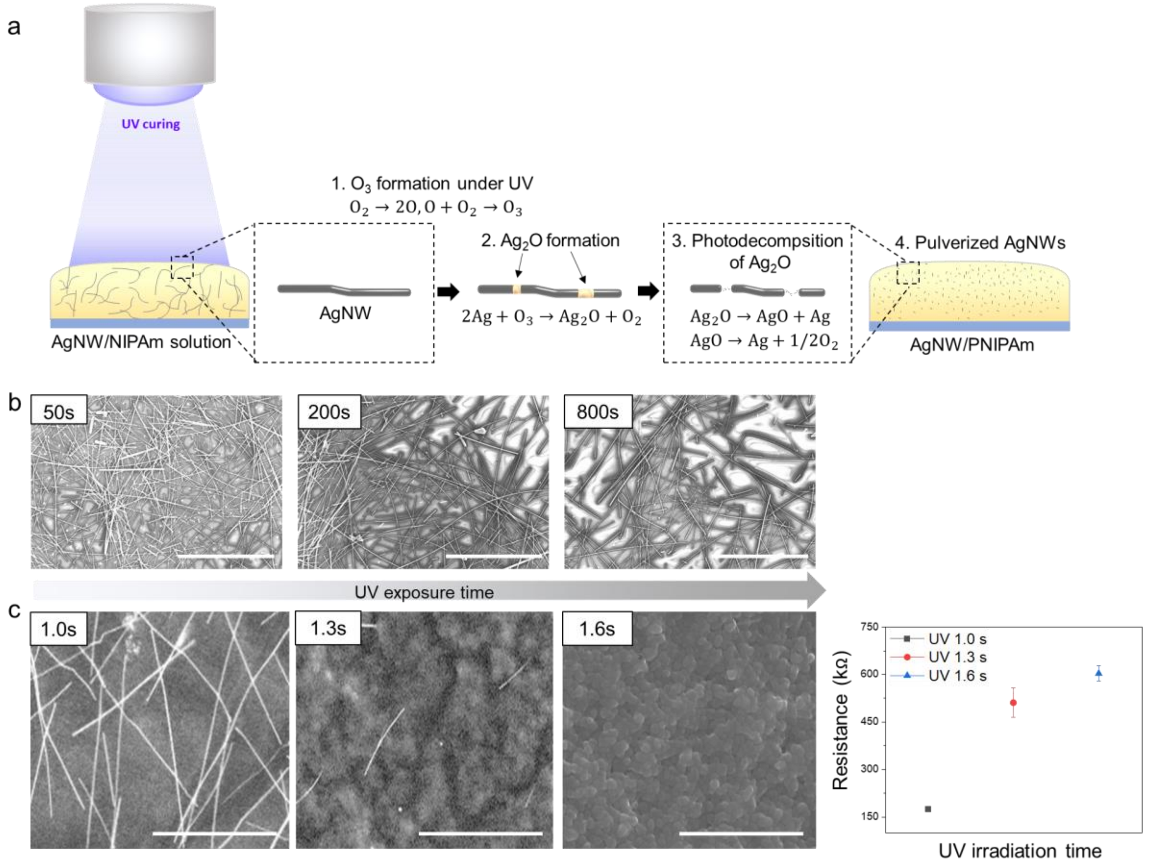


**Supplementary Figure 2. Decomposition of AgNWs during NIPAm polymerization.** (a) When polymerization of NIPAm solution with AgNWs is proceeding, more AgNWs are pulverized by increasing duration time of UV radiation. In the presence of $O_3$, an oxide layer composed of $Ag_2O$ was partially formed on AgNW by ultraviolet (UV)[23]. Even in the absence of $O_3$, this phenomenon still occurs due to the conversion of $O_2$ in the atmosphere to $O_3$ under UV during the polymerization of AgNW/NIPAm solutions. $Ag_2O$ is photo-sensitive and has an unstable property in a light irradiation environment, so it is decomposed into Ag and AgO by UV, and AgO is also unstable at room temperature and is converted to Ag and $O_2$[24,25]. (b) As the exposure time to UV increases, pristine AgNWs undergo photodecomposition by the reaction mentioned in (a), eventually becoming shorter AgNWs[26]. Scale bars, 10 µm. (c) AgNWs in AgNW/NIPAm solution become Ag metal debris, dispensed into the PNIPAm matrix. Scale bars, 5 µm (left). We also measured sheet resistance of each samples using 4-point probe method. A sample with 1.6s UV exposure time showed the highest electrical resistance due to pulverized AgNWs, while 1.0s presented relatively low resistance (right).

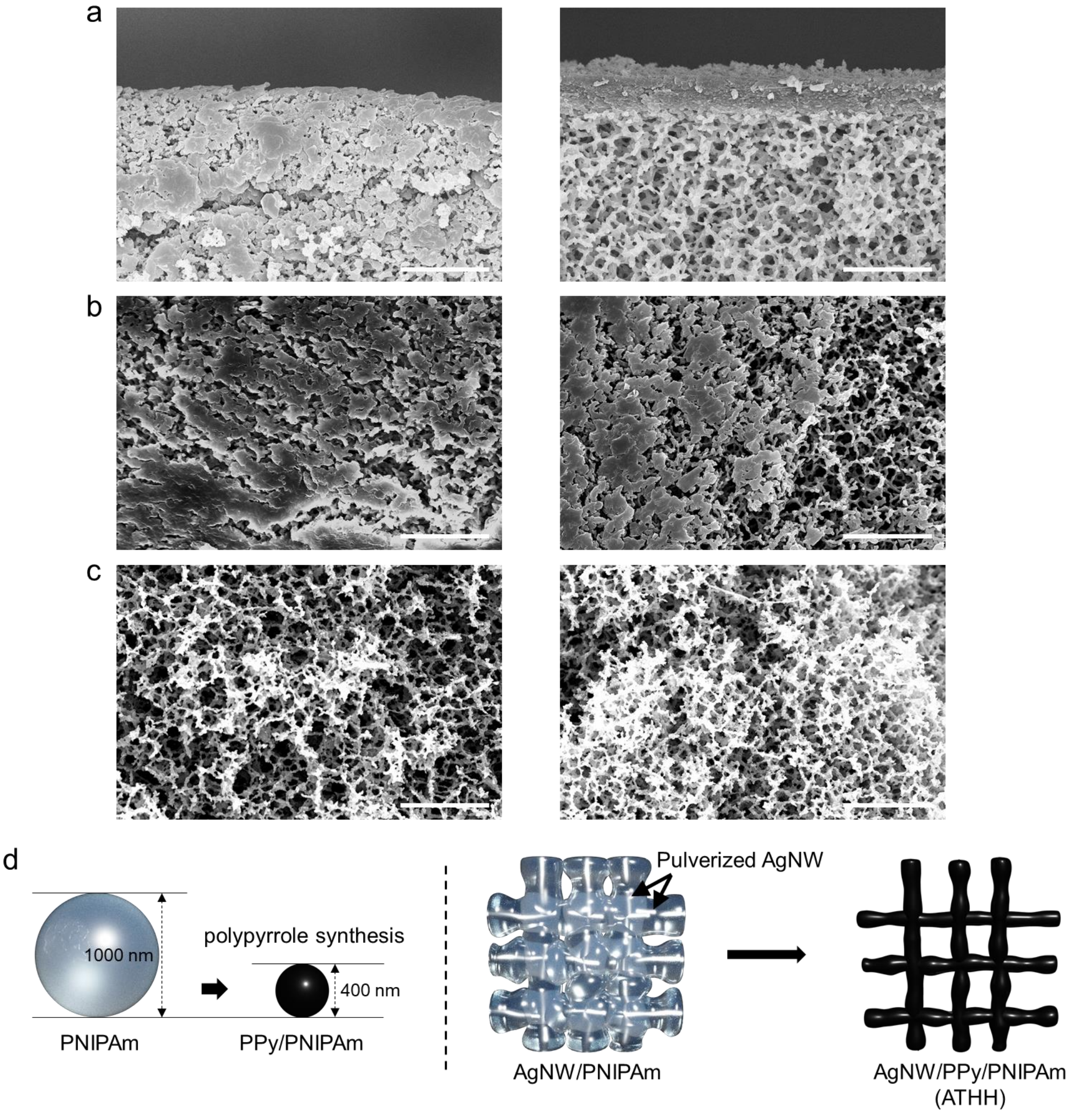


**Supplementary Figure 3. Cross-sectional SEM images of the hydrogel films.** (a) Cross-sectional images of ATHH synthesized using half concentration of pyrrole monomer and oxidant solutions (left) and same concentration of the reagents used in our paper (right). When the pyrrole monomer and $AgNO_3$ concentrations are half the values used in our paper, the porosity is low. However, when the concentrations are doubled, the size of the composite particles forming the network decreases and the porosity increases significantly. (b) ATHH synthesized with polypyrrole for 6 hours (left) and 12 hours (right) each. The ATHH synthesized for 6 hours shows a few pores, but the sample synthesized for 12 hours has more pores within the film due to the formation of a higher amount of PPy/PNIPAm composite particles with smaller sizes. (c) ATHH synthesized with AgNW solutions of 2-fold (left) and 4-fold concentrations (right), respectively. As the concentration of AgNW increases, the frames of the pores become denser, indicating that the main component forming this frame is AgNW. All samples were freeze-dried. Scale bars, 10 µm. (d) Schematic of pore formation in ATHH during polypyrrole synthesis.

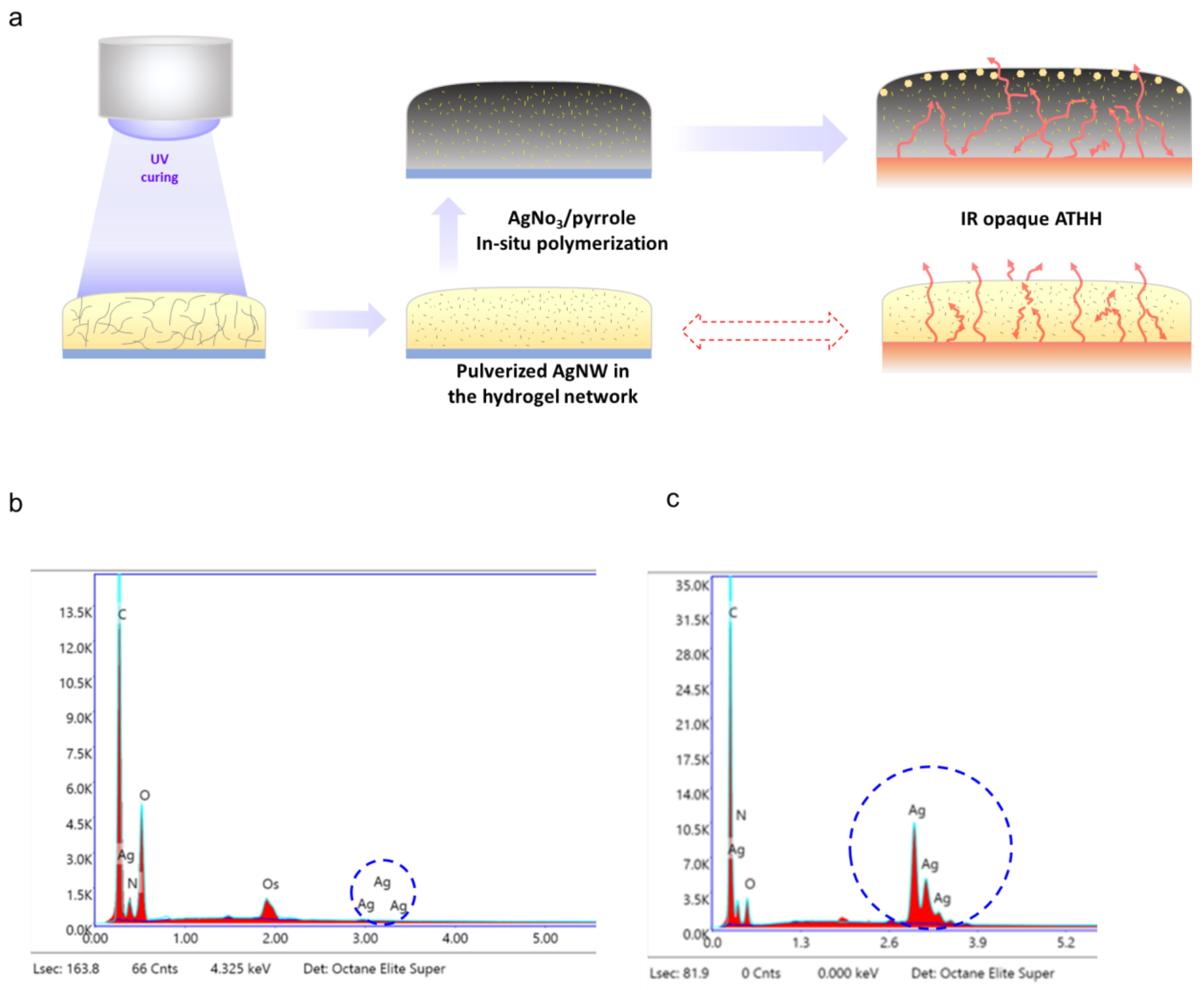


**Supplementary Figure 4. The Fabrication processes and surface energy-dispersive spectroscopy (EDS) data of ATHH.** (a) Schematics of fabrication processes for the ATHH. AgNW/PNIPAm film is cured by UV irradiation. Ag particles and polypyrrole are synthesized over AgNW/PNIPAm network which has pulverized AgNWs through $AgNO_3$ and polypyrrole in-situ polymerization. The heating effect of ATHH was caused by IR trapping due to IR absorbing and scattering within the ATHH network composed of merged polypyrrole and Ag particles (right). EDS spectra of (b) AgNW/PNIPAm and (c) ATHH.

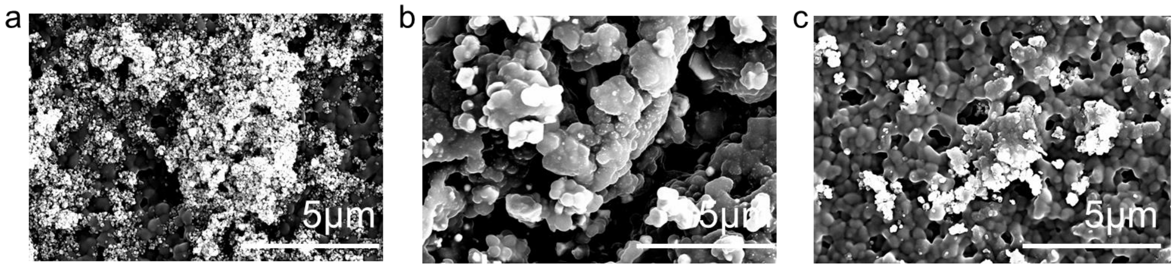


**Supplementary Figure 5. Surface SEM images of ATHH films synthesized with different surfactants.** ATHH films fabricated with 5 wt% of (a) Tween 80, (b) SDBS and (c) Triton X-100 during polypyrrole synthesis. Tween 80 was effective in controlling the size of spherical silver particles, but it was difficult to synthesize size particles near 3 μm. Triton X-100 and SDBS show negligible effect on particle size control.

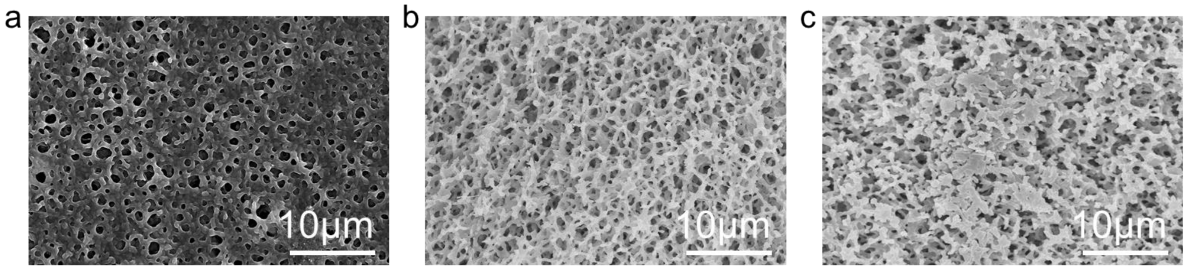


**Supplementary Figure 6. SEM images of freeze-dried ATHH films according to the concentration of reagents.** (a) Surface and (b) cross-sectional SEM images of ATHH films prepared with half concentration of reagents and (c) cross-sectional SEM image of doubled concentration of reagents used for polypyrrole synthesizing. When ATHH is synthesized using half or normal concentration of reagents, there are no pore blockages. However, ATHH prepared using doubled concentration of reagents used in our paper shows pore blockages, clogging the fluidic channels.

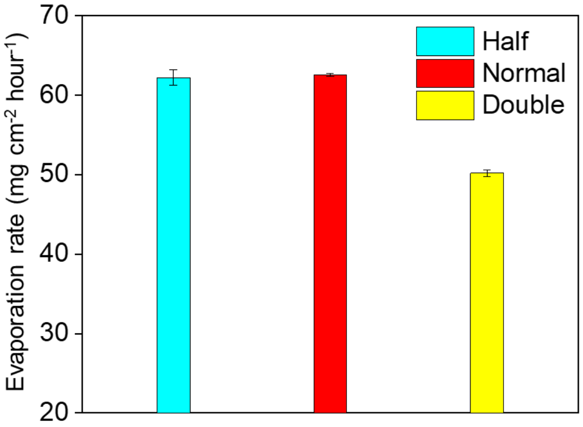


**Supplementary Figure 7. Evaporation rate depending on the concentration of reagents.** when the concentration of the reagents for a polypyrrole synthesis is doubled, water evaporation rate decreases compared to the samples prepared using same or lower concentration of reagents used in our paper.

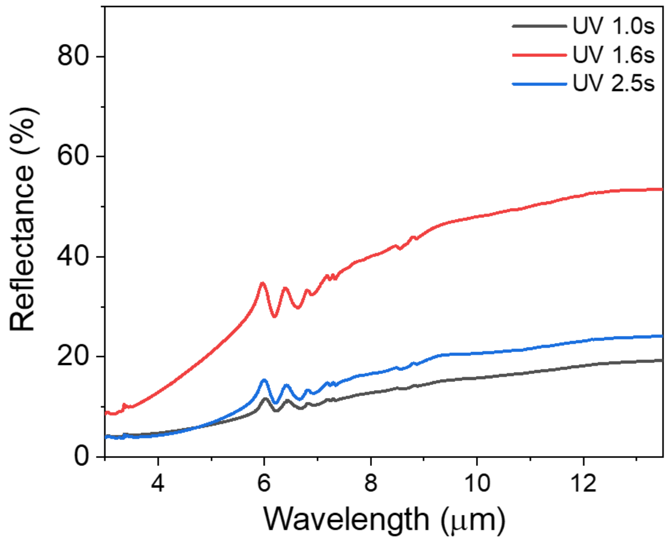


**Supplementary Figure 8. Reflectance spectra of AgNW/PNIPAm films with different UV radiation times during polymerization.** AgNW/PNIPAm films prepared with UV radiation time of 1.6 s shows the highest IR reflectance.

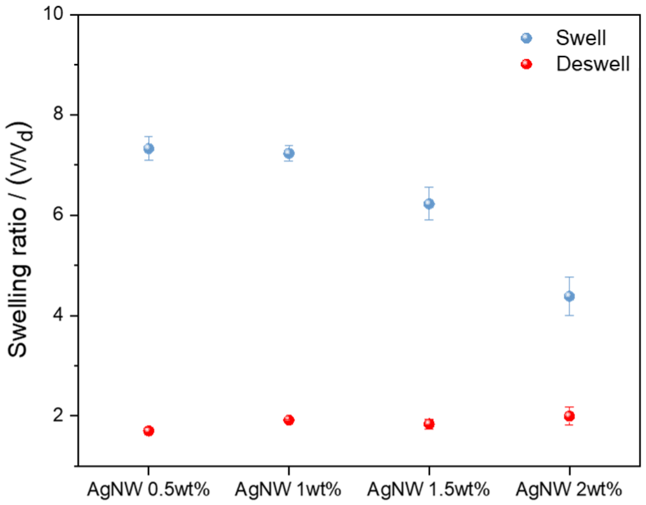


**Supplementary Figure 9. Swelling ratio of ATHH.** ATHH films prepared with different concentration of AgNWs. V is volume of a hydrated film and $V_d$ means volume of a fully dehydrated film. The volume of the hydrated films was measured at 20°C and 50°C for swollen and de-swollen state, respectively.

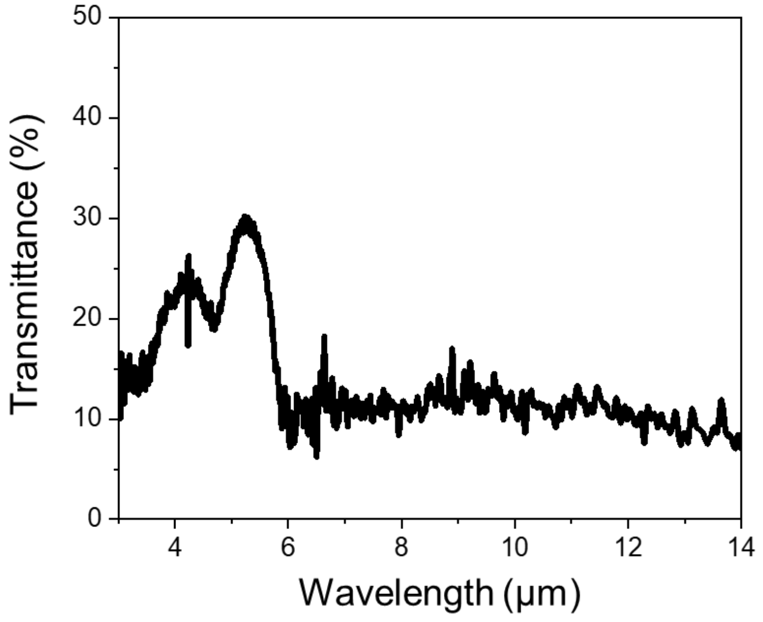


**Supplementary Figure 10. The Infrared transmittance spectra of polypyrrole coated PNIPAm.** In order to see the effect of only polypyrrole, the film was synthesized using iron (III) chloride hexahydrate as an oxidant that did not precipitate silver particles on the surface.

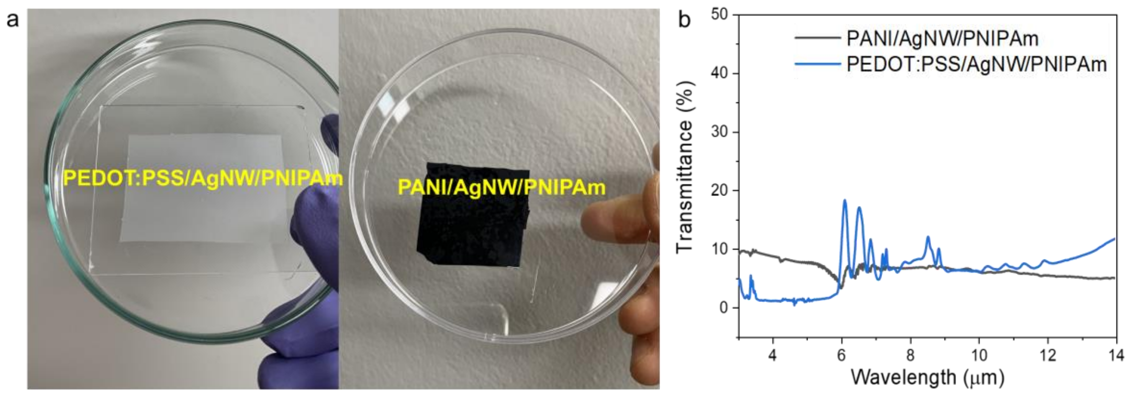


**Supplementary Figure 11. Synthesized hydrogel films using other conductive polymers.** (a) PNIPAm hydrogel films with embedded polyaniline (PANI) (left) and poly(3,4-ethylenedioxythiophene):poly(styrenesulfonate) (PEDOT:PSS) (right)). (b) Measured IR transmittance of the hydrogel films in (a).

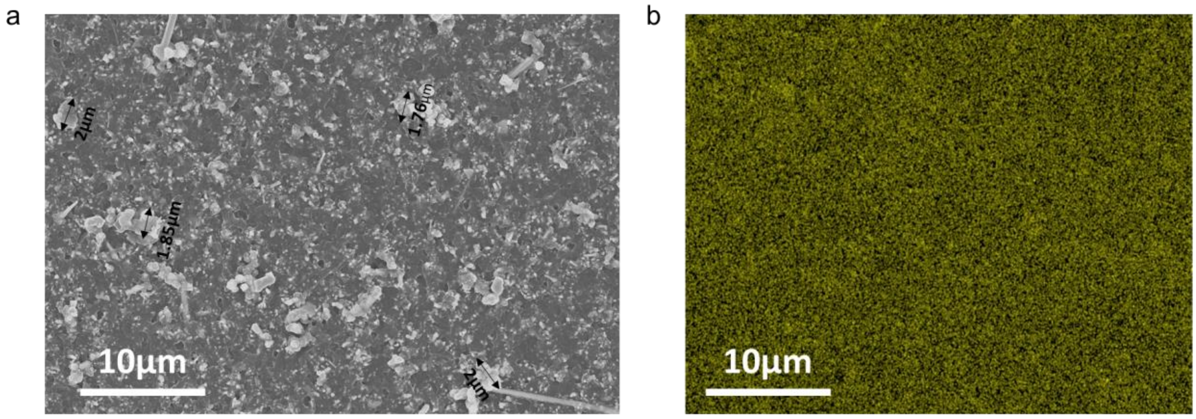


**Supplementary Figure 12. SEM image and EDS data of the ATHH (surface).** (a) SEM surface image of the ATHH and (b) elemental signal mapping of silver using energy-dispersive spectrometer in the area of (a).

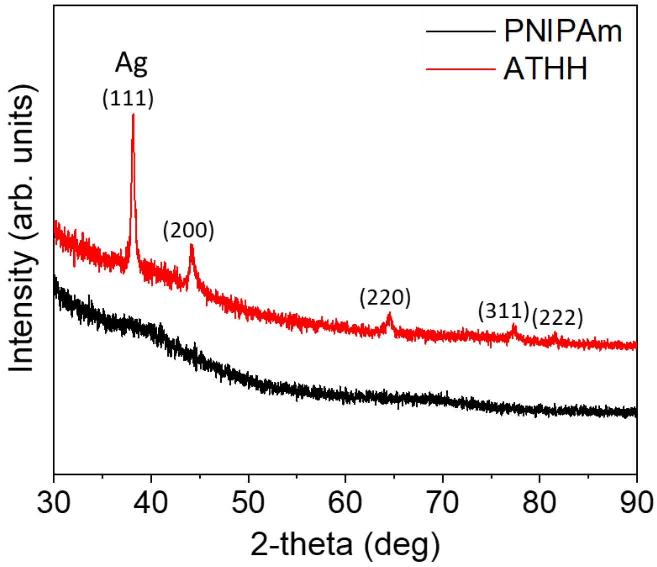


**Supplementary Figure 13. X-ray diffraction (XRD) data of the ATHH and PNIPAm.** ATHH shows XRD peaks perfectly matched with ICSD data of silver.

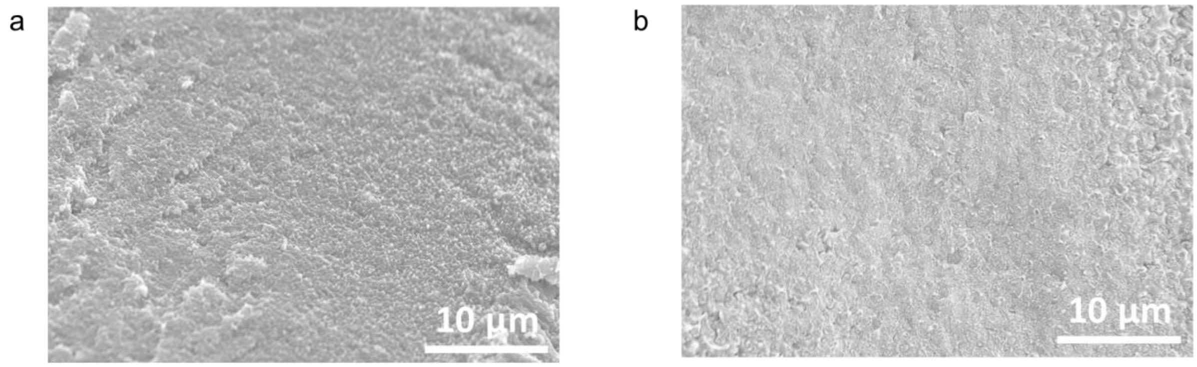


**Supplementary Figure 14. Cross-section image of air-dried PNIPAm and AgNW/PNIPAm.** Unlike in the ATHH, (a) PNIPAm shows densely packed structure due to the hydrogel undergoes equilibrium polymeric chain conformation due to slow water evaporation rate and becomes entangled due to Van der Waals interaction and hydrogen bonding[27]. (b) AgNW/PNIPAm also has a densely packed structure due to an absence of the reduction effect in the hydrogel particle size through the polypyrrole synthesis.

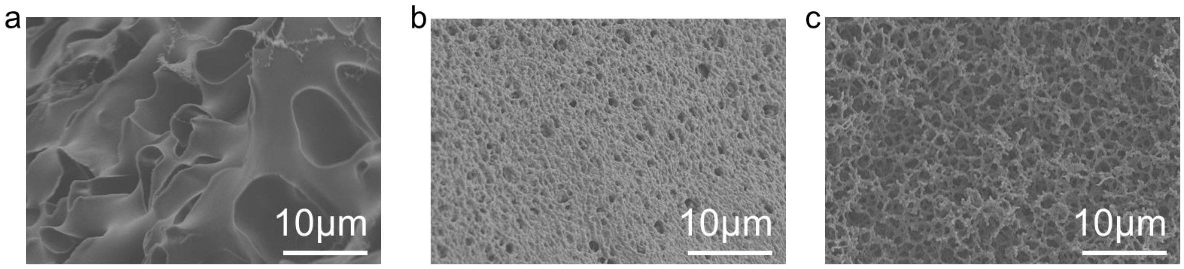


**Supplementary Figure 15. Cross-sectional SEM images of freeze-dried hydrogel films.** Freeze-dried (a) PNIPAm has the large pores that are not interconnected. The formation of ice crystals inside the hydrogel and the sublimation of these ice crystals results in large pores surrounded by a thick outer wall[28] that could interfere with the fluid flow. In the case of freeze-dried (b) AgNW/PNIPAm and (c) ATHH, they have similar morphology regardless of the drying method due to AgNW interfere with growth of big ice crystals and the change in conformation of the hydrogel polymer.

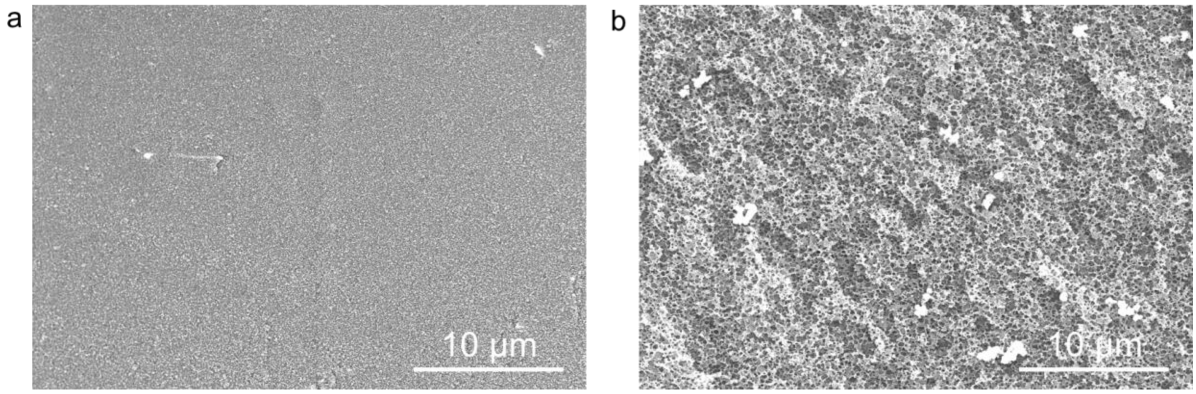


**Supplementary Figure 16. Cross-sectional SEM images of air-dried and freeze-dried ATHH films with a quarter AgNW concentration.** (a) Air-dried and (b) freeze-dried ATHH synthesized with AgNW solution of a quarter concentration. The air-dried sample showed densely packed structure due to the polymer entanglement, while the freeze-dried sample exhibited finely porous structure.

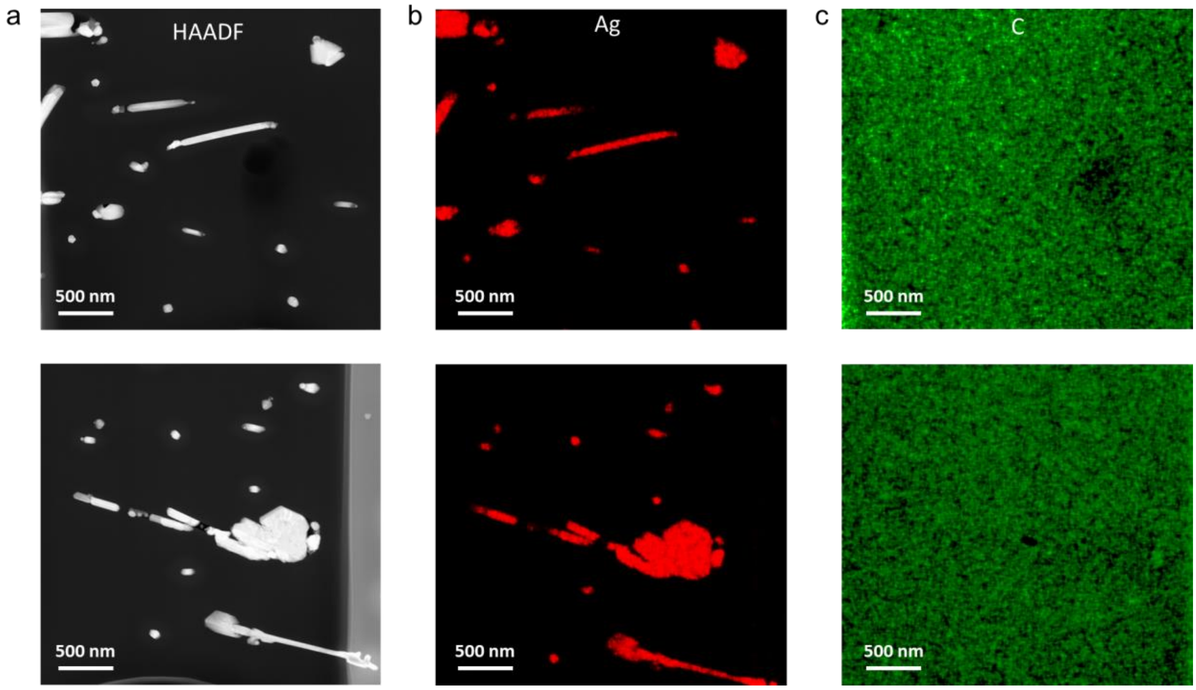


**Supplementary Figure 17. HAADF-STEM images and corresponding EDS data of ATHH.** (a) HAADF-STEM images of ATHH. TEM sample was prepared using Focused-Ion Beam machine. (b) Elemental signal mapping of silver using EDS mode. (c) EDS data of carbon in the ATHH. Those data mean silver particles are embedded within carbon-based hydrogel network.

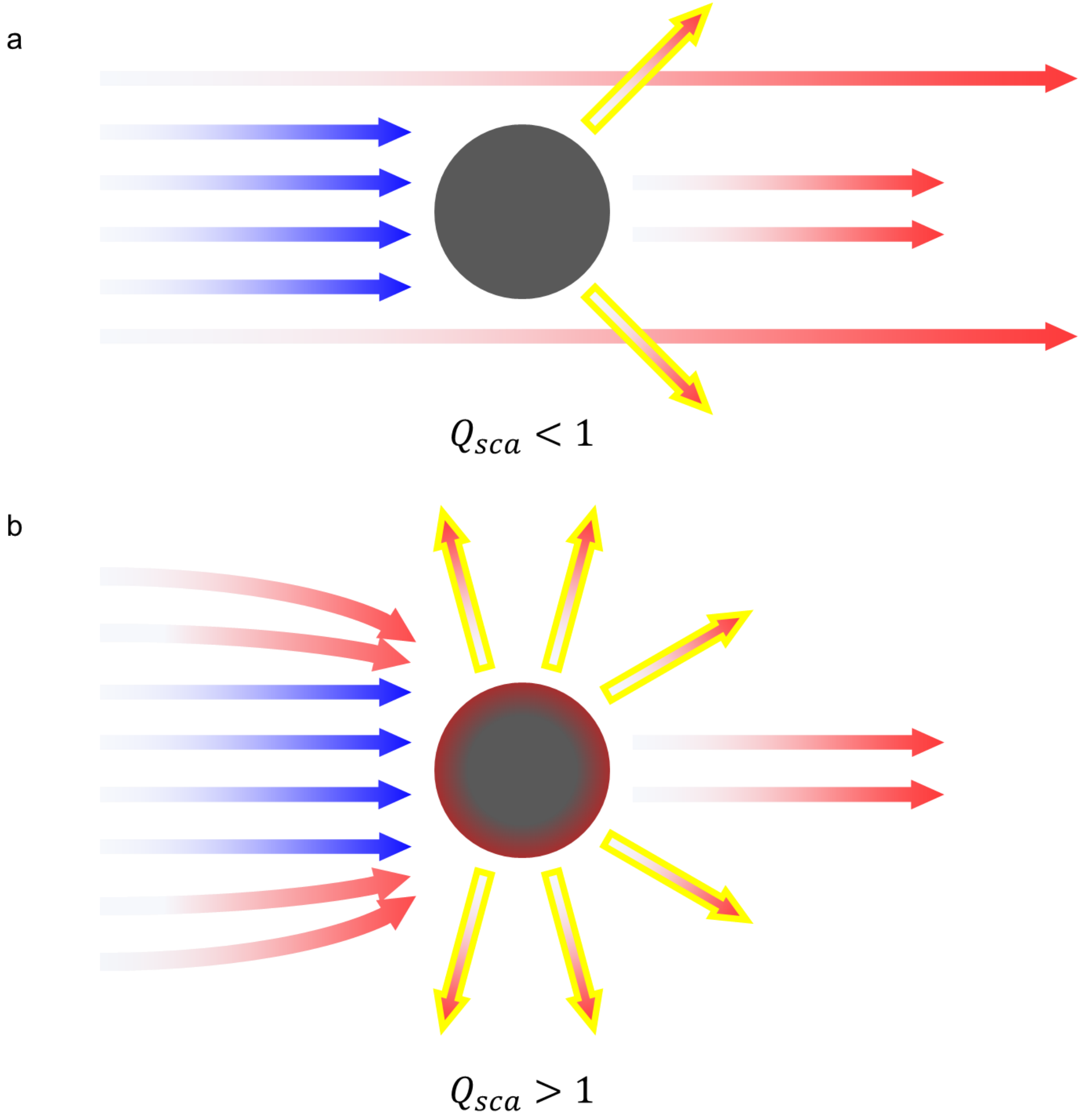


$$Q_{sca} \equiv \frac{\text{Scattered Energy}}{\text{Incident Energy}} = \frac{\text{Scattering Cross Section}}{\text{Geometrical Cross Section}}$$

**Supplementary Figure 18. Scattering efficiency of silver particles.** Scattering efficiency ($Q_{sca}$) is physically defined as scale factor between an energy of scattered light due to a particle (yellow-edged arrows) and an energy of incident light directing to cross-section of particle (green-edged arrows). In principle, those quantities can be represented as scattering cross-section (i.e., ratio between an energy of scattered light and an intensity of incident light) and geometrical cross-section, respectively. Please note that scattering phenomenon occurs in entire $4\pi$-solid-angle direction, including forward and backward direction of incoming light. (a) For a common particle without any or meaningful electromagnetic interaction, there is no interacting behavior of incident light and $Q_{sca}$ is less than unity. (b) If electromagnetic interaction occurs in a particle, incoming radiation, even neighboring light, can be interacted with the particle. Consequently, resulting amount of scattered energy can be larger than the energy of geometrically incident light, and corresponding $Q_{sca}$ can also be larger than unity.

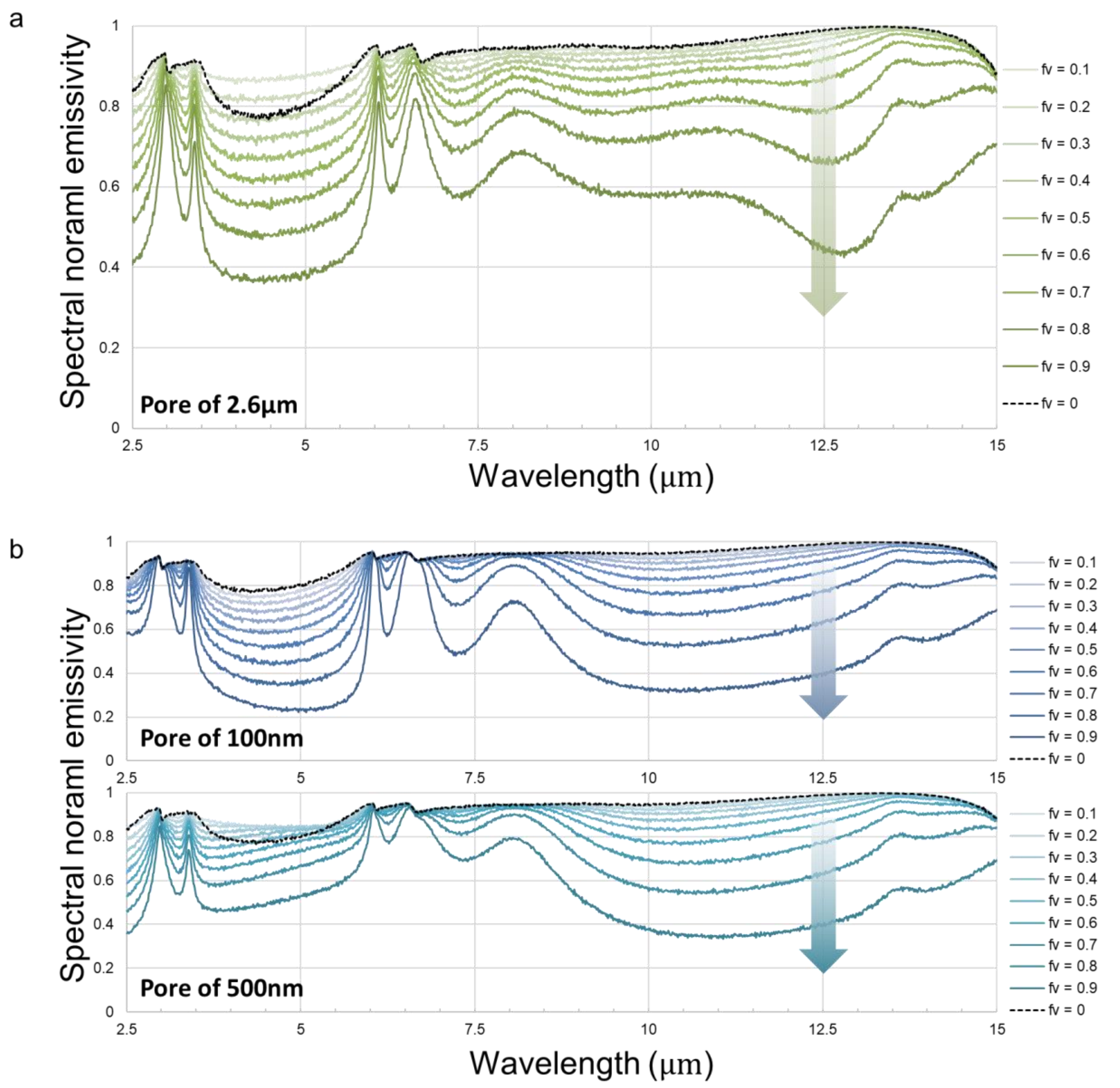


**Supplementary Figure 19. Spectral normal emissivity of ATHH varied with pore sizes.** Spectral normal emissivity of ATHH with pore size of (a) 2.6 μm and (b) nanometer-sized pores of 100 and 500 nm, respectively.

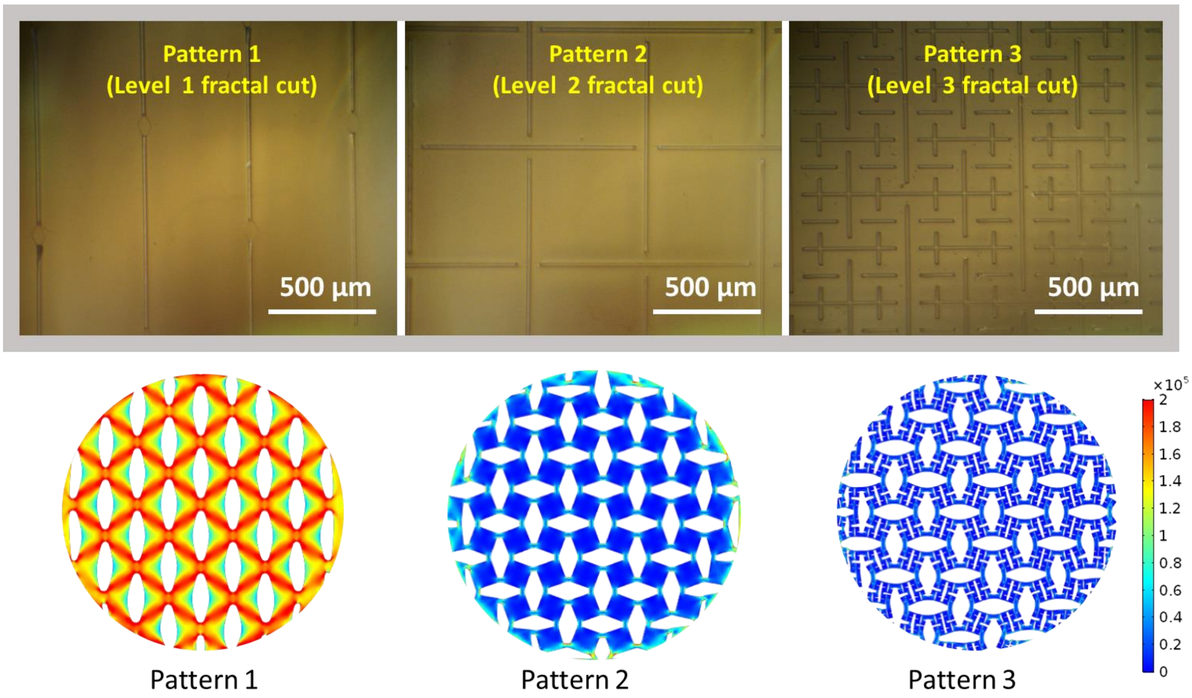


**Supplementary Figure 20. Simulated top view of ATHH with three different fractal patterns.** We conducted simulation using COMSOL Multiphysics. When the ATHH is thermally contracted, the maximum von mises stress from the lateral maximum strain of the pattern 1, 2, and 3 were 8.89×105, 2.85×105, and 1.90×105 Pa, respectively. The pattern 3 opens and closes most stably as the lowest von mises stress value.

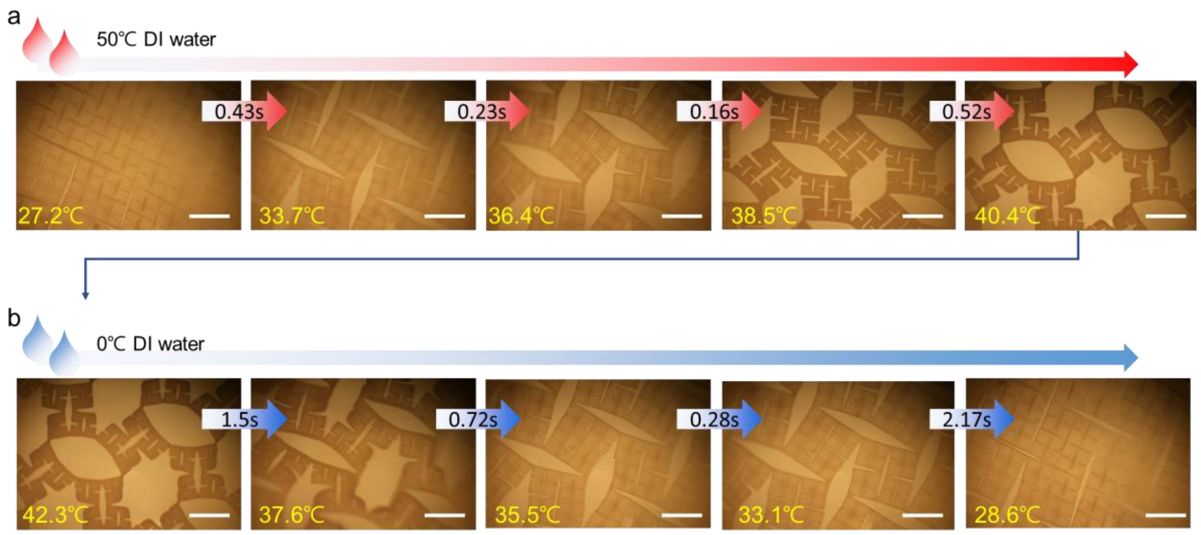


**Supplementary Figure 21. Images of open and closed auxetic pattern in ATHH.** a) Pouring hot water of 50°C into the ATHH in order to open the auxetic pattern and b) cold water of 0°C to close the pattern. Scale bars, 500 μm.

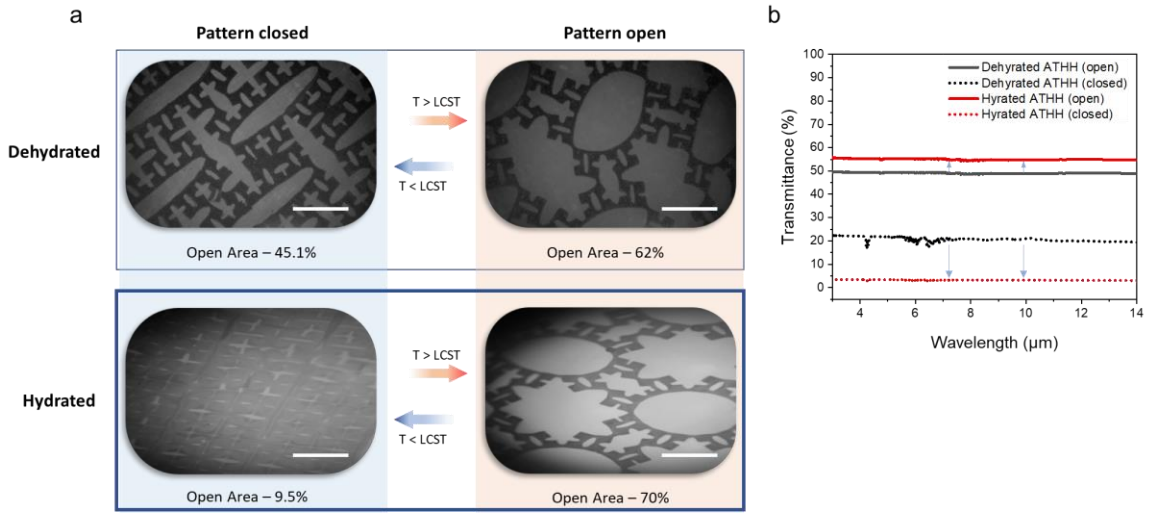


**Supplementary Figure 22. Calculated IR transmittance comparing fully dehydrated ATHH and hydrated ATHH.** a) Optical images of fully dehydrated ATHH when the pattern is close and open (top). Optical images of hydrated ATHH when the pattern is close and open (bottom). b) Measured transmittance of ATHH within IR range and calculated transmittance of ATHH based on the measured numerical value, adapting a space of the real image from optical microscope when the pattern is open and close. Scale bars, 500 μm.

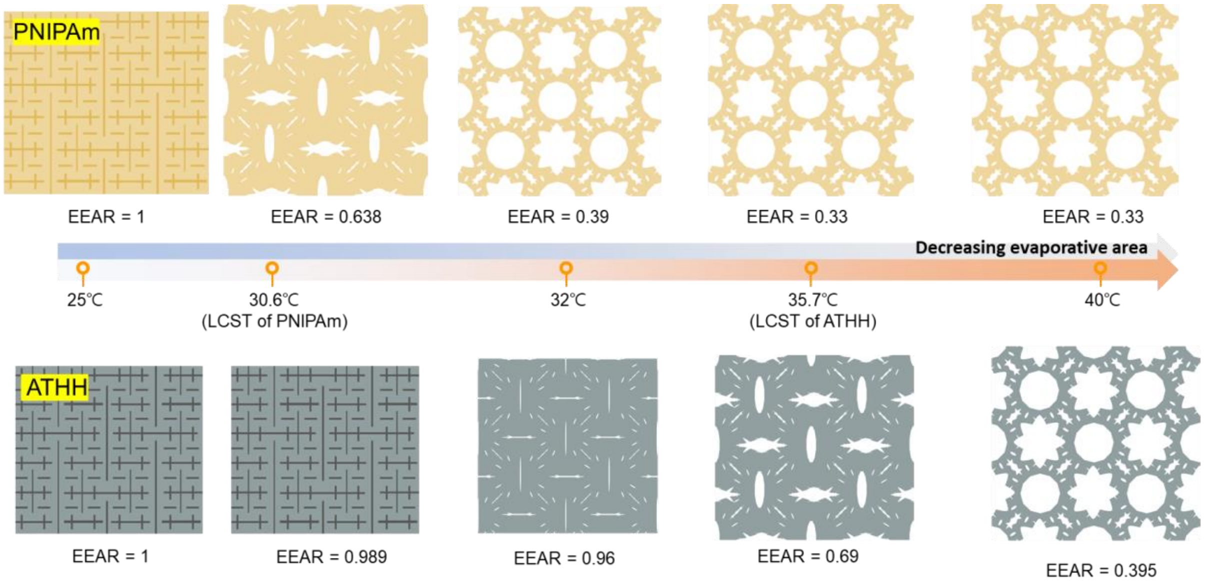


**Supplementary Figure 23. Schematic of effective evaporative area ratio (EEAR).** Increasing the temperature, PNIPAm and ATHH open the auxetic pattern, and EEAR gradually decreases. ATHH presented LCST of 35.7℃, therefore the evaporative area of ATHH where evaporation occurs gradually decreases over LCST compared to the PNIPAm, which enhances the cooling effect.

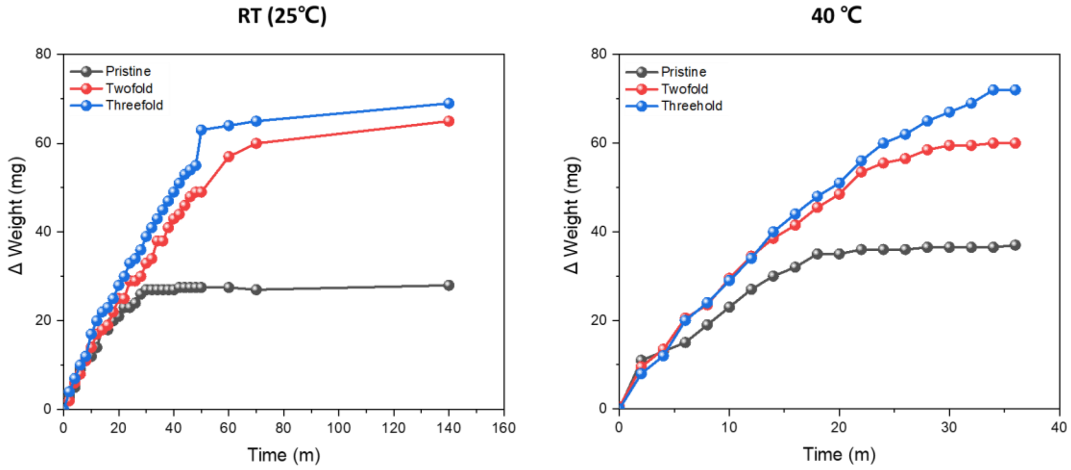


**Supplementary Figure 24. Absolute weight change of ATHH depending on the thickness at RT and 40 ℃ under dehydration.** We measured the weight of ATHH varying the thickness of ATHH to confirm the practical usage and reliability at each temperature.

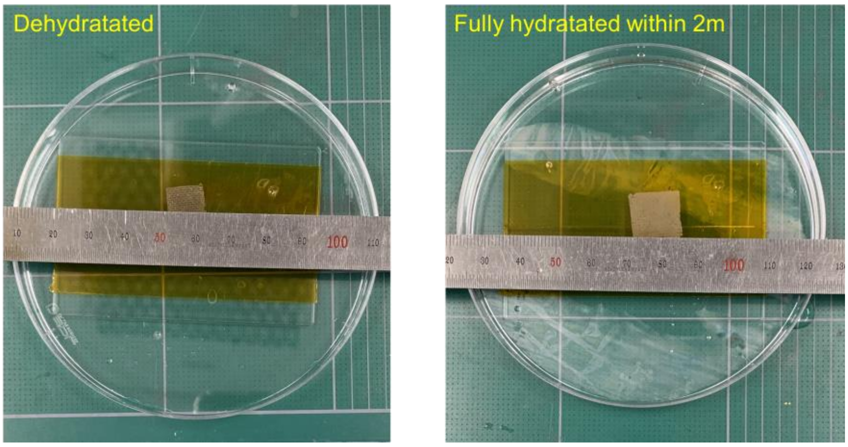


**Supplementary Figure 25. Hydration and dehydration states of patterned ATHH.** Patterned ATHH was dried on the hot plate for one hour to fully dehydrate the hydrogel, and DI water of 24°C was poured for the hydration.

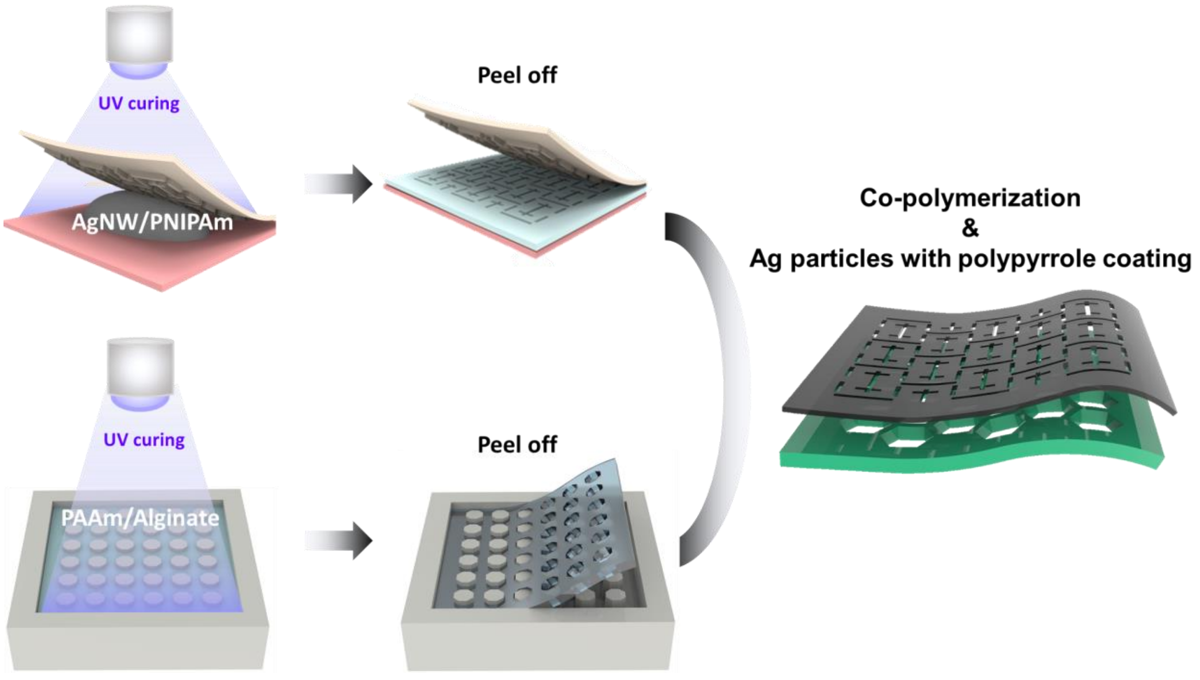


**Supplementary Figure 26. Fabrication steps for the S-ATHH combining with PAAm/Alginate as an substrate layer.** AgNW/PNIPAm film was cured by UV exposure, pressing auxetic-patterned PDMS stamp (top). PAAm/Alginate hydrogel was synthesized on a honeycomb-shaped sapphire mold (bottom). Both patterned AgNW/PNIPAm and PAAm/Alginate were co-polymerized through free radicals remained state under UV exposure, and coated with polypyrrole and $AgNO_3$ solution for enhanced thermal scattering.

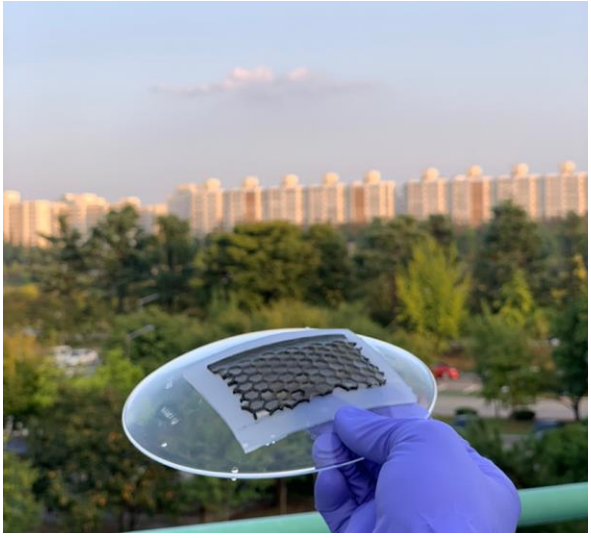

**Supplementary Figure 27. A photograph of S-ATHH for large area coverage.** On account of the soft lithography through the silicon etching process, the size of ATHH could be regulatable.

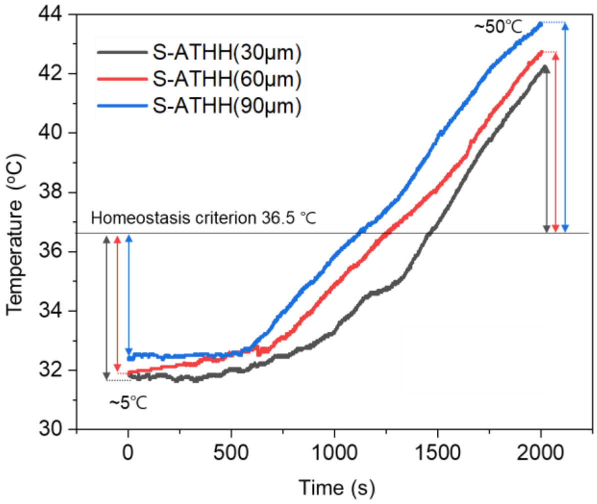


**Supplementary Figure 28. The effect of S-ATHH thicknesses for thermal balance.** The environmental temperature rose from 5℃ to 50℃, and the thermal balance of three types of S-ATHH thicknesses (30 μm/60 μm/90 μm) were measured.

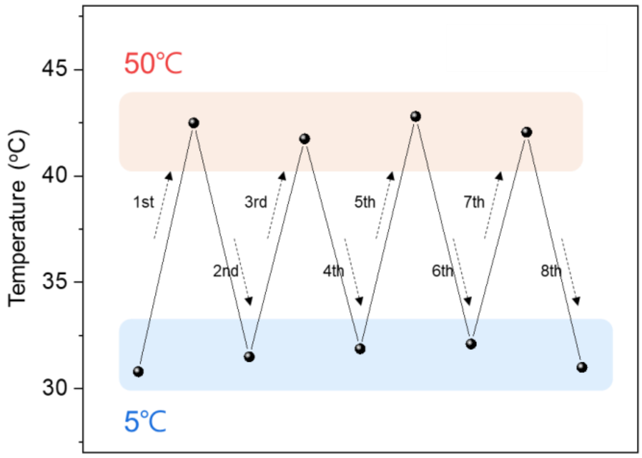


**Supplementary Figure 29. Bidirectional temperature controllability of reused ATHH.** The maximum temperature deviation of S-ATHH was measured at 5℃ and 50℃, respectively in the thermo-hygrostat.

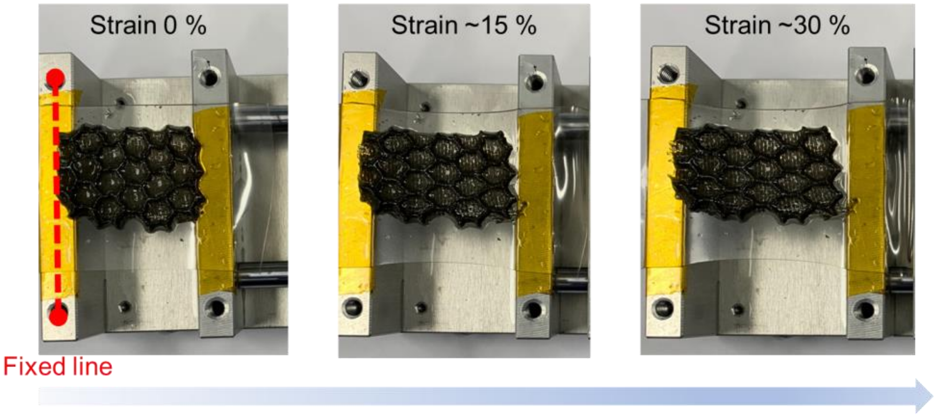


**Supplementary Figure 30. Mechanical durability test about stain stress of auxetic-patterned ATHH.** Patterned ATHH was firmly attached on the stretchable VHB film. We fixed VHB film on the left side and applied strain for the right side by a tensioning machine.

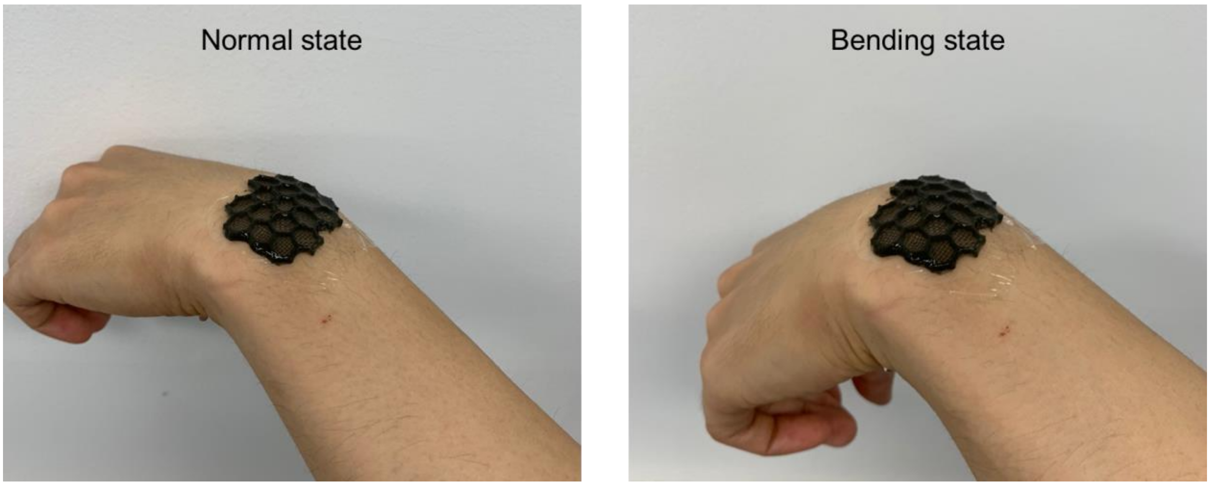


**Supplementary Figure 31. Conformability of auxetic-patterned ATHH on the human skin.** Patterned ATHH was fixed on the wrist with a 3M medical tape to create a seamless surface. Due to the auxetic pattern and stretchable substrate made of PAAm/Alginate, ATHH conformally attached even at the bent state.

**Supplementary references**


1 Modest, M. F. *Radiative Heat Transfer*. (Elsevier Inc., 2013).
2 Incropera, F. P., Dewitt, D. P., Bergman, T. L. & Lavine, A. *Principles of Heat and Mass Transfer*. (2013).
3 Jeon, J., Park, S. & Lee, B. J. Optical property of blended plasmonic nanofluid based on gold nanorods. *Opt Express* **22 Suppl 4**, A1101-1111 (2014).
4 Bohren, C. F. & Huffman, D. R. *Absorption and Scattering of Light by Small Particles*. (WILEY-VCH Verlag GmbH & Co. KGaA, 1998).
5 Waxenegger, J., Trügler, A. & Hohenester, U. Plasmonics simulations with the MNPBEM toolbox: Consideration of substrates and layer structures. *Computer Physics Communications* **193**, 138-150 (2015).
6 Seo, J., Qin, C., Lee, J. & Lee, B. J. Tailoring the Spectral Absorption Coefficient of aBlended Plasmonic Nanofluid Using a CustomizedGenetic Algorithm. *Sci. Rep.* **10**, 8891 (2020).
7 Raki´c, A. D., Djurisˇ ic, A. B., Elazar, J. M. & Majewski, M. L. Optical properties of metallic films for vertical-cavity optoelectronic devices. *Appl. Opt.* **37**, 5271-5283 (1998).
8 Z.M. Zhang, Nano/Microscale heat transfer, *McGraw-Hill Education* (2007).
9 W. Cai, *Optical Metamaterials*, Springer New York (2010).
10 Yalcın̦ *et al.* Colored Radiative Cooling Coatings with Nanoparticles. *ACS Photonics* **7**, 1312-1322, (2020).
11 Leung, E. M. *et al.* A dynamic thermoregulatory material inspired by squid skin. *Nat. Commun.* **10**, 1947 (2019).
12 Peng, Y. *et al.* Nanoporous polyethylene microfibres for large-scale radiative cooling fabric. *Nat. Sustain.* **1**, 105-112 (2018).
13 Hsu, P. C. *et al.* A dual-mode textile for human body radiative heating and cooling. *Sci. Adv.* **3**, e1700895 (2017).
14 Cheng, Y. *et al.* Highly Stretchable and Conductive Copper Nanowire Based Fibers with Hierarchical Structure for Wearable Heaters. *ACS Appl. Mater. Interfaces* **8**, 32925-32933 (2016).
15 Hong, S. *et al.* Wearable thermoelectrics for personalized thermoregulation. *Sci. Adv.* **5**, eaaw0536 (2019).
16 Cai, L., Song, A.Y., Wu, P. et al. Warming up human body by nanoporous metallized polyethylene textile. *Nat. Commun.* **8**, 496 (2017).
17 Ehrmann, A. & Blachowicz T. Thermal Properties of Textiles. *In Examination of Textiles Mathematical and Physical Methods*, 113-123 (Springer, 2017).
18 Hashan, M. M. et al. Functional properties improvement of sock items using different types of yarn. *Inter. J. Text. Sci*. **6**, 34-42 (2017).
19 Majumdar, A., Mukhopadhyay, S. & Yadav, R. Thermal properties of knitted fabrics made from cotton and regenerated bamboo cellulosic fibres. *Int. J. Therm. Sci.* **49**, 2042-2048 (2010).
20 Prakash, C. & Ramakrishnan, G. Study of thermal properties of bamboo/cotton blended single jersey knitted fabrics. Arab. *J. Sci. Eng.* **39**, 2289-2294 (2014).
21 Hu, R., Yang, J., Yang, P. et al. Fabrication of ZnO@Cotton fabric with anti-bacterial and radiation barrier properties using an economical and environmentally friendly method. *Cellulose* **27**, 2901–2911 (2020).
22 Qi, B., Wang, F., Chen, Q. et al. Enzymatic construction of a temperature-regulating fabric with multiple heat-transfer capabilities. *Cellulose* **29**, 3513–3528 (2022).

23 Choo, D. C. & Kim, T. W. Degradation mechanisms of silver nanowire electrodes under ultraviolet irradiation and heat treatment. *Sci. Rep.* **7**, 1696 (2017).

24 Wang, X., Li, S., Yu, H., Yu, J. & Liu, S. Ag2O as a new visible-light photocatalyst: self-stability and high photocatalytic activity. *Chemistry* **17**, 7777-7780, (2011).

25 Kim, J.-H., Ma, J., Jo, S., Lee, S. & Kim, C. S. Enhancement fo antibacterial properties of a silver nanowire film via electron beam irradiation. *ACS Appl. Bio Mater.* **3**, 2117−2124 (2020).

26 Lin, C.-C., Lin, D.-X. & Lin, S.-H. Degradation problem in silver nanowire transparent electrodes caused by ultraviolet exposure. *Nanotechnology*, **31**, 215705 (2020).

27 Stanciu, L., Diaz-Amaya, S. *Introductory Biomaterials: An Overview of Key Concepts* (Elsevier Inc., 2021)

28 Simoni, R. C. et al. Effect of drying method on mechanical, thermal and water absorption properties of enzymatically crosslinked gelatin hydrogels. *An. Acad. Bras. Cienc.* **89**, 745-755 (2017).
