# Supplementary material for: Bidirectional thermo-regulating hydrogel composite for autonomic thermal homeostasis
Source: Nat Commun. 2023 May 26;14:3049. doi: 10.1038/s41467-023-38779-w (PMC10220006; doi:10.1038/s41467-023-38779-w)
Supplement: Supplementary file 3 — Description of Additional Supplementary Files [file 41467_2023_38779_MOESM3_ESM.pdf]

Description of Additional Supplementary Files

File Name: Supplementary Movie 1
Description: Real-time monitoring of auxetic patterned ATHH during thermal fluctuation.
